# Disrupted Iron Metabolism and Mortality during Co-infection with Malaria and an Intestinal Gram-Negative Extracellular Pathogen

**DOI:** 10.1016/j.celrep.2020.108613

**Published:** 2021-01-12

**Authors:** Luara Isabela dos Santos, Thais Abdala Torres, Suelen Queiroz Diniz, Ricardo Gonçalves, Gustavo Caballero-Flores, Gabriel Núñez, Ricardo Tostes Gazzinelli, Kevin Joseph Maloy, Lis Ribeiro do V. Antonelli

**Affiliations:** 1 Instituto René Rachou, Fundação Oswaldo Cruz, Belo Horizonte 30190-009, Minas Gerais, Brazil; 2 Sir William Dunn School of Pathology, University of Oxford, South Parks Road, Oxford OX1 3RE, UK; 3 Instituto de Ciências Biológicas, Departamento de Bioquimica e Imunologia, Universidade Federal de Minas Gerais, Belo Horizonte 31270-901, Minas Gerais, Brazil; 4 University of Massachusetts Medical School, Worcester, MA 01605-2324, USA; 5 Departamento de Patologia Geral, ICB, Universidade Federal de Minas Gerais, Belo Horizonte, Minhas Gerais, Brazil; 6 Department of Pathology and Rogel Cancer Center, University of Michigan Medical School, Ann Arbor, MI 48109, USA; 7 Institute of Infection, Immunity and Inflammation, University of Glasgow, Sir Graeme Davies Building, 120 University Place, Glasgow G12 8TA, Scotland

**Keywords:** malaria, *Plasmodium*, co-infection, Gram-negative bacteria, mortality, *Citrobacter rodentium*, hemolysis, iron, heme

## Abstract

Individuals with malaria exhibit increased morbidity and mortality when infected with Gram-negative (Gr−) bacteria. To explore this experimentally, we performed co-infection of mice with *Plasmodium chabaudi* and *Citrobacter rodentium*, an extracellular Gr− bacterial pathogen that infects the large intestine. While single infections are controlled effectively, co-infection results in enhanced virulence that is characterized by prolonged systemic bacterial persistence and high mortality. Mortality in co-infected mice is associated with disrupted iron metabolism, elevated levels of plasma heme, and increased mitochondrial reactive oxygen species (ROS) production by phagocytes. In addition, iron acquisition by the bacterium plays a key role in pathogenesis because co-infection with a mutant *C. rodentium* strain lacking a critical iron acquisition pathway does not cause mortality. These results indicate that disrupted iron metabolism may drive mortality during co-infection with *C. rodentium* and *P. chabaudi* by both altering host immune responses and facilitating bacterial persistence.

## Highlights

• Co-infection with malaria and a Gram-negative bacterial pathogen leads to high mortality
• Co-infection leads to elevated plasma heme and systemic bacterial persistence
• Iron acquisition is critical for bacterial persistence and mortality

## Graphical Abstract

**Figure:**
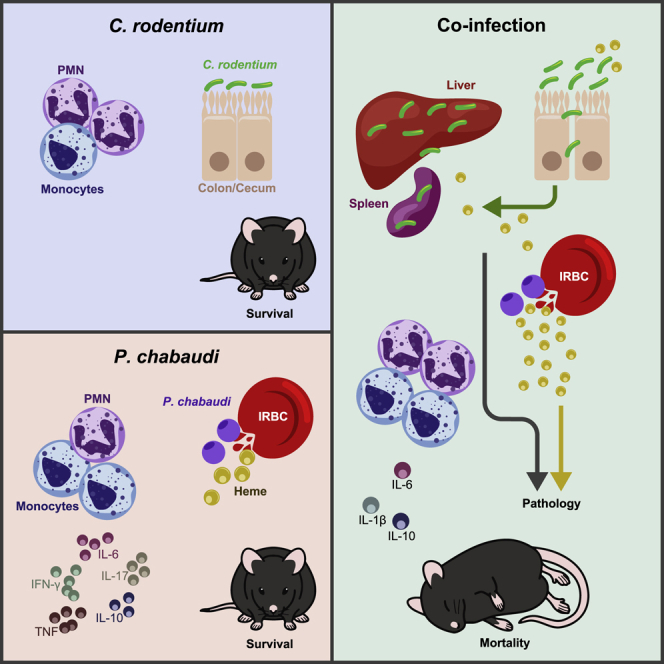

## Teaser

Mortality of individuals with malaria and bacteremia is higher than with malaria alone. dos Santos et al. report that co-infection with *Citrobacter rodentium* and *Plasmodium chabaudi* results in high mortality that is associated with increased plasma heme, sustained bacterial persistence, and altered host immunity.

## Introduction

Human infectious diseases exhibit a skewed geographical distribution, and co-infection with different pathogens is common in the developing world (Brooker and Clements, 2009; McShane, 2005; Scott et al., 2011). Intestinal bacteria not only shape local immunity but also impact systemic immune activation, and this may have either protective or detrimental effects on disease (Kamada et al., 2013), but the impact of intestinal infection on susceptibility to other infectious diseases is poorly understood. However, epidemiological data indicate that individuals in malaria-endemic regions exhibit increased morbidity and mortality when infected with Gram-negative (Gr−) bacterial pathogens (Berkley et al., 1999; Scott et al., 2011; Were et al., 2011). Indeed, the mortality of patients with malaria and bacteremia is up to eight times higher than in individuals with malaria alone (Were et al., 2011). Studies in sub-Saharan Africa reported that pediatric cases of invasive non-typhoidal *Salmonella* (NTS) infections exhibited the same seasonal variation patterns as malaria infection (Feasey et al., 2015; Mabey et al., 1987) and that children who are heterozygous for the sickle cell trait that protects against severe malaria also exhibit reduced risk of Gr− bacteremia (Scott et al., 2011). Although NTS is the most frequent culprit identified in malaria-infected patients with Gr− bacteremia, pathogenic strains of *Escherichia coli* have been identified in ∼5%–10% of cases (Berkley et al., 1999; Davenport et al., 2016; Ezenwa et al., 2018; Nielsen et al., 2015; Park et al., 2016; Were et al., 2011). However, the precise cellular and molecular mechanisms through which concomitant Gr− bacterial infections drive excessive mortality remain unclear.

Experimental studies have utilized co-infection of mice with malaria and the intracellular pathogen *S. enterica* to mimic acute NTS infection. Malaria pre-infection was associated with increased *Salmonella* colonization and impaired control of systemic NTS infection (for review, see Mooney et al., 2019). Several mechanisms have been implicated in defective control of systemic *Salmonella* infection in malaria-infected mice, especially impaired function of phagocytes, which play a major role in protection from NTS (for review, see Mooney et al., 2019). Myeloid cell defects have been associated with increased interleukin-10 (IL-10) levels and induction of heme oxygenase-1 (HO-1) during malaria infection, leading to deficient granulocyte mobilization, reduced reactive oxygen species (ROS) production, and impaired bacterial killing (Cunnington et al., 2011, 2012; Lokken et al., 2014). However, it is not known whether similar mechanisms account for the increased susceptibility of malaria-infected individuals to extracellular Gr− bacterial pathogens, such as pathogenic *E. coli* strains.

Infection of mice with the Gr− enteric bacterial pathogen *Citrobacter rodentium* is used as a model of human diarrheagenic *E. coli* infections, particularly enterohemorrhagic or enteropathogenic *E. coli* (EHEC/EPEC) infections (Collins et al., 2014; Mundy et al., 2005). Oral inoculation with *C. rodentium* leads to attachment and colonization of colonic epithelial cells and effacement of brush border microvilli, termed attaching and effacing (A/E) lesions, as well as colonic hyperplasia and mild intestinal inflammation (Collins et al., 2014; Mundy et al., 2005). Like most diarrheagenic *E. coli* infections, *C. rodentium* usually causes self-limiting, localized, and transient disease that is controlled by innate and adaptive immune mechanisms, which clear the pathogen ∼3 weeks post-infection (Collins et al., 2014; Mundy et al., 2005). To define potential mechanisms responsible for exacerbated disease during co-infection with an extracellular Gr− enteric pathogen, we combined experimental malaria infection of mice with *Plasmodium chabaudi* sp. (AS) (*P. chabaudi*) (Langhorne et al., 2004; Stevenson et al., 1992) and concomitant infection with *C. rodentium*. We show that while single infections with either pathogen were controlled effectively, co-infection led to very high levels of morbidity and mortality, due to prolonged systemic persistence of the bacterial pathogen. Mortality was not driven by excessive host inflammatory responses, but it was associated with altered iron homeostasis and elevated plasma heme concentrations in co-infected mice. We also show that phagocytes from co-infected mice exhibit increased ROS production and that iron acquisition is essential for bacterial persistence. Thus, disrupted iron metabolism may drive mortality during co-infection through effects on both host immune responses and by aiding pathogen persistence.

## Results

### High Mortality in Mice Co-infected with *P. chabaudi* and *C. rodentium*

**Figure 1:**
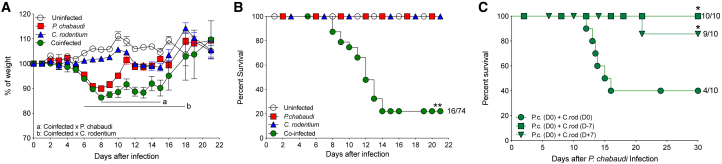
High Mortality in Mice Co-infected with *P. chabaudi* and *C. rodentium* (A and B) C57BL/6 mice were infected intraperitoneally with 1 × 10^6^*P. chabaudi*-RBCs or orally with 1 × 10^9^*C. rodentium* or co-infected with both at day 0. (A) Body weights were measured daily. Symbols represent group means ± SEM as a percentage of the initial body weight. Regions “a” and “b” show at least p < 0.05 between indicated groups. (B) Survival was monitored up to day 21. Data are pooled from six independent experiments (n = 26 for uninfected controls; n = 42 for single infection groups; n = 74 for co-infected group). (C) Cohorts of C57BL/6 mice that were infected with *P. chabaudi*-RBCs at day 0 were inoculated orally with *C. rodentium* at day 0, or 1 week before *P. chabaudi* infection (D-7), or 1 week after *P. chabaudi* infection (D+7) and survival was monitored until day 30. Results are representative of two independent experiments (n = 5–10 mice per group). Statistical significance was determined by either two-way ANOVA (A) or Mantel-Cox (B and C) test (^∗^p < 0.05 and ^∗∗^p < 0.01).

To determine the impact of intestinal infection on morbidity and mortality in a murine malaria model, we compared four groups of C57BL/6 mice treated as follows: (1) single infection with *P. chabaudi* using intraperitoneal inoculation of infected red blood cells (*P. chabaudi*-RBCs); (2) single infection with *C. rodentium* via gavage; (3) co-infection with *P. chabaudi* and *C. rodentium*; and (4) uninfected controls. Following single infection with *C. rodentium*, mice exhibited transient diarrhea and weight loss during the second week of infection but recovered between 14 and 21 days post-infection (dpi) (Figure 1A). Single infection of mice with *P. chabaudi* led to a more severe weight loss, followed by recovery in the third week of infection (Figure 1A). Although the co-infected group exhibited comparable weight loss during the first week as mice with single *P. chabaudi* infection, most co-infected mice did not recover and had significantly lower weights during the second week of co-infection (Figure 1A). We consistently observed high levels of mortality (60%–90%) in co-infected cohorts during the second week of co-infection, whereas both single infection groups showed 100% survival (Figure 1B). To examine whether concomitant co-infection was important for mortality, we staggered the time of co-infection, giving the *C. rodentium* infection either 1 week before or after the *P. chabaudi* infection. We found that mortality was only induced by contemporaneous co-infection (Figure 1C). These results show that co-infection with malaria and an extracellular Gr− enteric bacterial pathogen results in high mortality, validating this model as a tool to investigate potential mechanisms involved in the increased mortality observed in malaria-endemic regions upon co-infection with Gr− bacteria.

### *P. chabaudi* Disease Kinetics Was Not Altered by *C. rodentium* Co-infection

**Figure 2:**
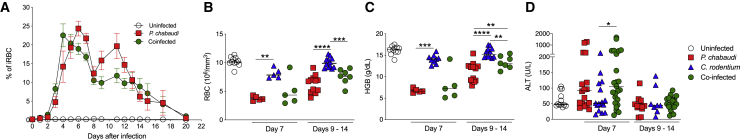
*P. chabaudi* Infection Kinetics Was Not Altered by *C. rodentium* Co-infection (A) C57BL/6 mice were infected with *P. chabaudi*-RBCs or/and with *C. rodentium*. Parasitemia was determined. Symbols represent group means ± SEM of pooled data from five independent experiments (n = 20 for uninfected controls; n = 35 for *P. chabaudi* infection group; n = 70 for co-infected group). (B–D) On 7 days post-infection (dpi) and throughout the period of mortality (days 9–14), co-infected mice were culled whenever they became moribund, and on the same day animals from the other groups were also culled to provide time-matched comparator samples from all groups. (B) RBC numbers and (C) hemoglobin (HGB) levels were assayed in heparinized blood samples, and (D) ALT levels were measured in serum samples. (B and C) Data represent pooled data from three independent experiments (n = 5–12 mice/group/time point). (D) Data represent pooled results from four independent experiments (n = 8–28 mice/group/time point). Each symbol represents an individual mouse and bars denote group means. Statistical significance was determined by the Mann-Whitney test (^∗^p < 0.05, ^∗∗^p < 0.01, and ^∗∗∗^p < 0.001).

Parasitemia following *P. chabaudi* infection typically reaches 20%–50% of infected RBCs ∼7 dpi, dropping to a sub-patent level after 3–4 weeks (Stephens et al., 2012). Although there was a trend of slightly earlier onset of primary parasitemia in co-infected mice, this was not observed across experiments and the peak parasitemias were similar in single and co-infected groups (Figure 2A). As hemolytic anemia during malaria is associated with poor prognosis (Alexandre et al., 2010; Castro-Gomes et al., 2014; Lamikanra et al., 2007), we analyzed RBC counts and hemoglobin levels during the primary phase of parasitemia (day 7 post-infection) and during the period of mortality in the co-infected group (days 9–14 post-infection). Single infection with *P. chabaudi* led to severe decreases in RBC counts and hemoglobin levels that mirrored the parasitemia kinetics (Figures 2B and 2C). Co-infected mice exhibited comparable reductions in RBC counts and hemoglobin levels as single *P. chabaudi*-infected mice at both time points evaluated (Figure 2B and 2C). As malaria infection is associated with pronounced changes in the liver (Rocha et al., 2015), we assayed serum levels of alanine aminotransferase (ALT) as a surrogate of liver damage. We found that single *P. chabaudi* infection or co-infection induced a comparable, transient elevation of ALT at 7 dpi that returned to normal during the second week of infection (Figure 2D). Taken together, these results indicate that co-infection with *C. rodentium* has no significant impact on the kinetics or severity of *P. chabaudi* infection, suggesting that the observed mortality was not due to more severe manifestations of malaria infection.

### Co-infected Mice Harbor Higher *C. rodentium* Burdens but Intestinal Pathology Is Not Exacerbated

**Figure 3:**
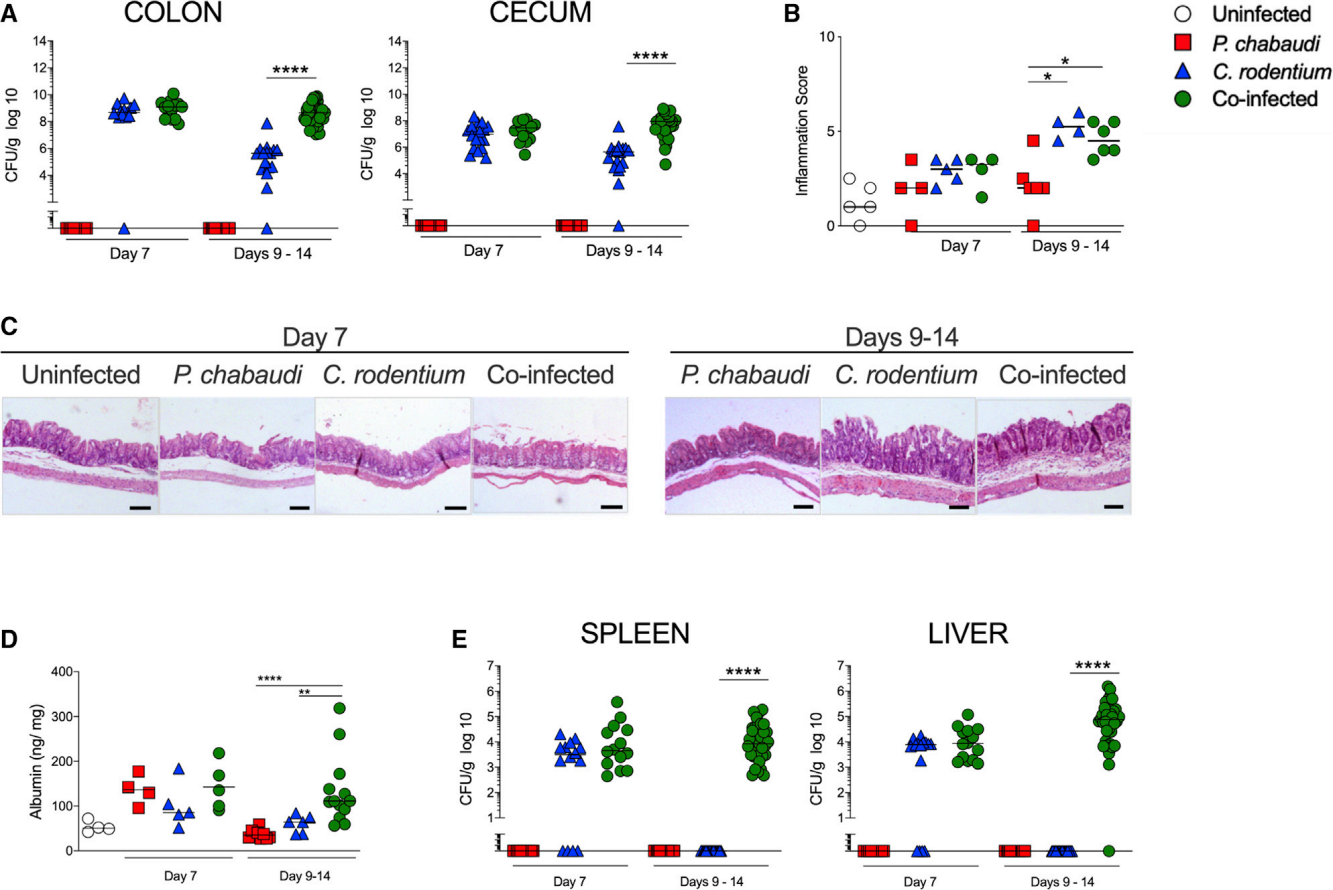
Co-Infected Mice Exhibit Higher Intestinal and Systemic *C. rodentium* Burdens (A) C57BL/6 mice were infected with *P. chabaudi*-RBCs and/or with *C. rodentium*. On 7 dpi and throughout the period of mortality (days 9–14), colon, cecum, spleen and liver samples were harvested for further analyses. *C. rodentium* colonization was determined by plating homogenized tissue samples on agar containing nalidixic acid. Each symbol represents an individual mouse and bars denote group means. Results are representative of four independent experiments (n = 12–36 mice/group/time point). (B) Cecum samples were fixed and then stained with hematoxylin and eosin. The inflammation scores were then evaluated. Each symbol represents an individual mouse and bars denote group means. Results are representative of three independent experiments (n = 4–6 mice/group/time point). (C) Representative photomicrographs depicting hematoxylin and eosin staining of cecum (magnification, ×50). Scale bar, 100 μm. (D) Albumin levels in intestinal contents were assayed by ELISA. Each symbol represents an individual mouse, and bars denote group means. Results are representative of two independent experiments (n = 4–12 mice/group/time point). (E) *C. rodentium* colonization was determined by plating homogenized tissue samples on agar containing nalidixic acid. Each symbol represents an individual mouse and bars denote group means. Data shown are representative of four experiments (n = 12–36 mice/group/time point). Statistical significance was determined by Mann-Whitney test (^∗^p < 0.05, ^∗∗^p < 0.01, and ^∗∗∗∗^p < 0.0001).

We next assessed whether mortality in co-infected mice was associated with impaired control of enteric bacterial infection. We found comparable levels of *C. rodentium* colonization in the ceca and colons of single *C. rodentium*-infected and co-infected mice at 7 dpi (Figure 3A). However, we observed significantly higher intestinal *C. rodentium* loads in *P. chabaudi* co-infected mice during the period of mortality (9–14 dpi) compared with single *C. rodentium*-infected mice (Figure 3A). The few co-infected mice that survived beyond 14 dpi exhibited clearance of *C. rodentium* by 21 dpi (Figure S1A). As expected (Mundy et al., 2005), single infection with *C. rodentium* led to mild intestinal inflammation with limited crypt hyperplasia and leukocyte infiltration (Figures 3B and 3C). Despite harboring higher bacterial burdens at later infection stages, we observed comparable mild intestinal pathology in co-infected mice (Figures 3B and 3C and S1B). Consistent with the low levels of intestinal pathology, single infection with *C. rodentium* had little impact on intestinal barrier integrity (Figure 3D). By contrast, single infection with *P. chabaudi* led to a transient and mild increase in intestinal albumin levels on 7 dpi, and this was not exacerbated by co-infection (Figure 3D). However, during the period of mortality, co-infected mice continued to exhibit elevated levels of intestinal albumin (Figure 3D). Overall, these results show that while malaria co-infection does not exacerbate intestinal inflammation, it leads to impaired clearance of an enteric pathogen and to a prolonged, minor defect in barrier function.

### Malaria Co-infection Leads to Increased Systemic Infection with *C. rodentium*

The prolonged colonization and elevated intestinal barrier permeability observed in co-infected mice prompted us to examine systemic tissues for evidence of infection. Comparable levels of viable *C. rodentium* were present in the spleen and liver at 7 dpi in *C. rodentium* single-infected mice and in mice co-infected with *P. chabaudi* (Figure 3E). However, while *C. rodentium* single-infected mice had no evidence of systemic colonization in the second week of infection, co-infected mice exhibited sustained systemic infection with *C. rodentium* (Figure 3E). Thus, co-infection with malaria leads to sustained systemic infection with an enteric Gr− pathogen throughout the period of peak mortality.

### Co-infection Was Associated with Reduced Local and Systemic Inflammatory Cytokines

**Figure 4:**
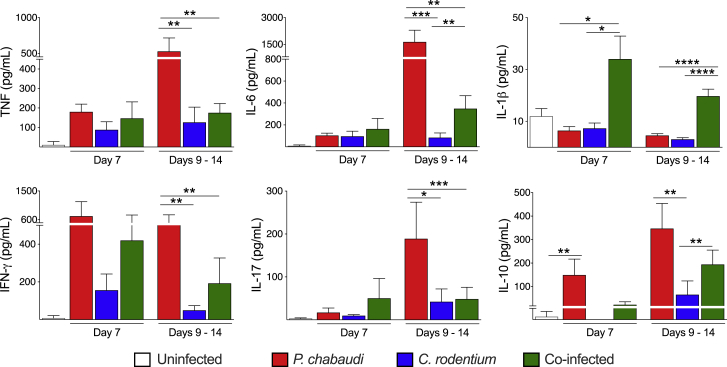
Co-infection with *C. rodentium* Reduces Systemic Inflammatory Cytokine Responses Compared with Single Infection with *P. chabaudi* C57BL/6 mice were infected with *P. chabaudi*-RBCs and/or with *C. rodentium*. On 7 dpi and throughout the period of mortality (days 9–14), serum samples were collected and cytokine levels were assayed. Bars represent group means ± SEM. Data shown are representative of two independent experiments (n = 4–17 mice/group/time point). Statistical significance was determined by Mann-Whitney test (^∗^p < 0.05, ^∗∗^p < 0.01, ^∗∗∗^p < 0.001, and ^∗∗∗∗^p < 0.0001).

Malaria pathogenesis is multi-factorial, and many symptoms can be linked to the systemic inflammation induced by the parasite (for reviews, see Clark et al., 2006; Gazzinelli et al., 2014). We hypothesized that bacterial co-infection might exacerbate the host inflammatory response during malaria, leading to a lethal “cytokine storm.” We observed that single *C. rodentium* infection had little impact on plasma cytokine levels, whereas single *P. chabaudi* infection increased plasma levels of tumor necrosis factor (TNF), IL-6, interferon-γ (IFN-γ), and IL-17 during the second week of infection (Figure 4). However, rather than enhancing these responses, co-infection with *C. rodentium* led to significant blunting of the plasma pro-inflammatory cytokine responses associated with *P. chabaudi* single infection (Figure 4). Although plasma IL-1β was significantly increased at 7 dpi in co-infected mice, these levels were very low and they decreased during the period of mortality (Figure 4). The anti-inflammatory cytokine IL-10 also showed a comparable increase in plasma in single *P. chabaudi*-infected and co-infected mice (Figure 4). A different pattern of cytokine production was observed in intestinal samples, with *C. rodentium* single infection leading to a modest increase in pro-inflammatory cytokines (TNF, IL-6, IFN-γ, and IL-17) and IL-10 in the colon and cecum (Figure S2). By contrast, single *P. chabaudi* infection led to comparable increases in TNF, IL-6, and IL-10 in the colon, but it did not affect cytokine production in the cecum (Figure S2). However, co-infection resulted in blunting of the local cytokine responses induced by *C. rodentium* in the colon and cecum (Figure S2). Together, these results indicate that cytokine storm was not responsible for the observed mortality in co-infected mice.

### Mortality during Co-infection Was Not Dependent on Key Innate Immune Pathways

**Figure 5:**
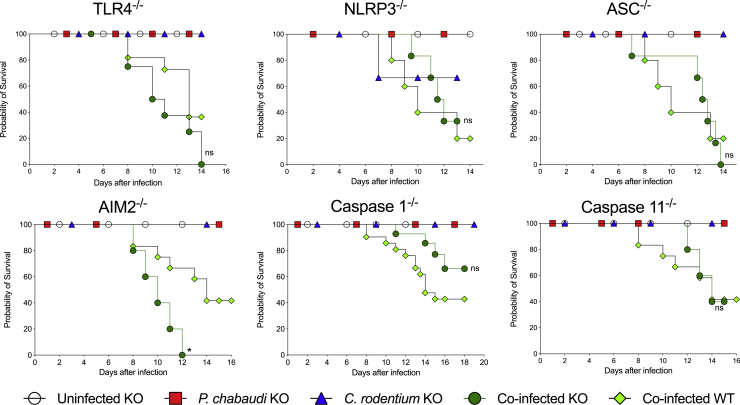
Mortality during Co-infection Was Not Dependent on Key Innate Immune Pathways Cohorts of mice lacking the indicated innate immune genes and control C57BL/6 mice were infected with *P. chabaudi*-RBCs and/or with *C. rodentium*, and survival was monitored. Results represent data from two independent experiments (n = 3–21 mice per group for each genotype). Statistical significance was determined by Mantel-Cox test comparing each co-infected knockout (KO) group with WT co-infected group from the same experiment (^∗^p < 0.05). ns, non-significant.

A previous study reported that murine malaria infection led to increased susceptibility to lethality following systemic administration of lipopolysaccharide (LPS) (Franklin et al., 2009), which triggers septic shock through activation of innate immune sensors, especially Toll-like receptor 4 (TLR4) and caspase-11 (Ataide et al., 2014; Franklin et al., 2009, 2011). To investigate whether innate immune receptor pathways were driving mortality during co-infection, cohorts of TLR4^−/−^, NLRP3^−/−^, ASC^−/−^, AIM2^−/−^, caspase-1^−/−^, and caspase-11^−/−^ mice were infected with *P. chabaudi* and/or *C. rodentium* and compared with concurrent co-infected wild-type (WT) controls. Although there was some minor inter-experimental variation, we found no significant differences in mortality between any of the innate immune receptor-deficient strains and the concurrent WT controls after co-infection (Figure 5). These results further demonstrate that mortality during co-infection was not due to hyperactivation of host inflammatory responses.

### Co-infected Mice Do Not Exhibit Defects in Phagocyte Mobilization or Function

**Figure 6:**
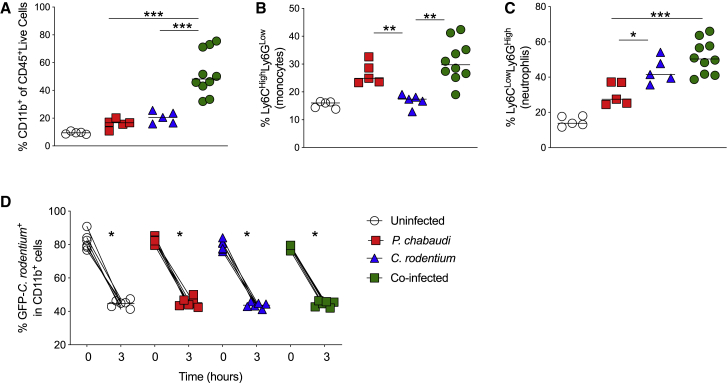
Co-infection Does Not Alter Recruitment or Phagocytic Function of Host Myeloid Cells (A–C) C57BL/6 mice were infected with *P. chabaudi*-RBCs and/or with *C. rodentium*. Spleens were harvested between 9 and 14 days post-infection. The proportions of (A) CD11b+ cells among live CD45+ leukocytes and (B) Ly6Ch^igh^Ly6G^int^ (monocytes) and (C) Ly6C^int^Ly6G^high^ (neutrophils) among live CD45+ CD11b^+^ cells were assessed by flow cytometry. Each symbol represents an individual mouse and bars denote group means. Data shown are representative of two independent experiments (n = 5-10 mice per group). (D). Spleen cells were cultured for 1 hour with GFP-*C. rodentium* and then with 1 hour with gentamicin. Cell suspensions were washed, and frequencies of GFP-*C. rodentium*^+^ CD11b^+^ cells were assessed at that (time 0h) and after additional 3 h of culture (time 3h). Each symbol represents an individual mouse and bars denote group means. Data shown are representative of two independent experiments (n = 6–7 mice/group). Statistical significance was determined by Mann-Whitney test (A and B) or Wilcoxon paired test (C), ^∗^p < 0.05, ^∗∗^p < 0.01, and ^∗∗∗^p < 0.001.

Phagocytes play a critical role in the control of both malaria and bacterial infections (Cunnington et al., 2011, 2012). During acute malaria infection in humans, monocytes are important both in the systemic inflammatory response and for parasite control (Antonelli et al., 2014; Sponaas et al., 2009). Conversely, neutrophils are critical during responses against extracellular bacterial infection (Sherman et al., 2018). In addition, studies with *Salmonella* and malaria co-infection have implicated impaired neutrophil function in the defective clearance of pathogenic bacteria (Cunnington et al., 2011). We therefore phenotypically and functionally analyzed splenic phagocyte populations (Figure S3). We observed a significant increase in the frequency of CD11b^+^ cells in co-infected mice relative to the single-infected groups (Figure 6A). The proportion of monocytes (Ly6C^high^Ly6G^int^) in co-infected mice was significantly higher than in *C. rodentium* single-infected mice but comparable with that observed in *P. chabaudi* single-infected mice (Figure 6B). Conversely, we found higher frequencies of neutrophils (Ly6C^int^Ly6G^high^) in both *C. rodentium* single-infected and co-infected mice than in *P. chabaudi* single-infected mice (Figure 6C). To functionally assess phagocytosis, we evaluated the uptake and killing of GFP-*C. rodentium* by CD11b^+^ splenocytes. We found similarly efficient uptake and destruction of *C. rodentium* by CD11b^+^ splenocytes in all groups (Figure 6D). These results suggest that the impaired control of *C. rodentium* in malaria co-infected mice was not due to a general impairment in systemic phagocyte mobilization or function.

### Co-infection Leads to Elevated Levels of Plasma Heme and Favors Bacterial Persistence

**Figure 7:**
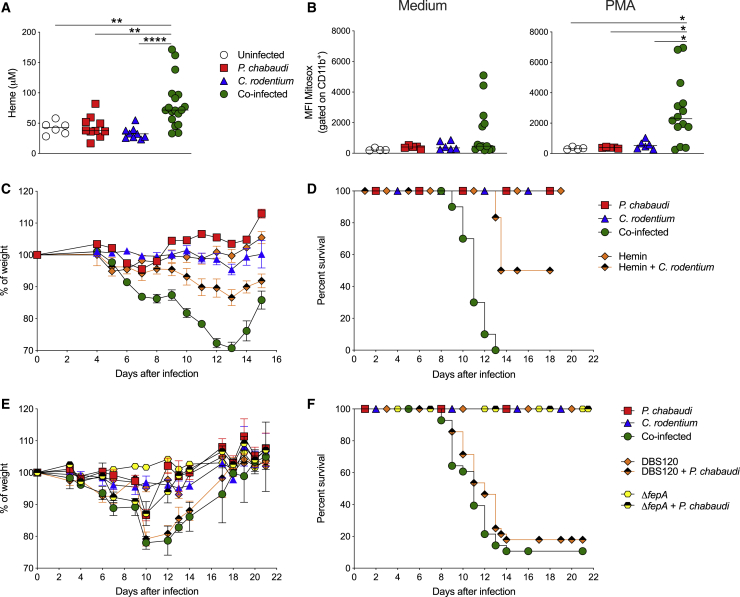
Co-infection Leads to Elevated Levels of Plasma Heme and Favors Bacterial Persistence Dependent on Uptake of Extracellular Iron (A) C57BL/6 mice were infected with *P. chabaudi*-RBCs and/or with *C. rodentium*. (A) Heme levels were measured in plasma samples collected between 9 and 14 days post-infection. Data represent pooled results from two independent experiments (n = 3–9 mice/group/time point). Each symbol represents an individual mouse and bars denote group means. Statistical significance was determined by the Mann-Whitney test. ^∗∗^p < 0.01 and ^∗∗∗∗^p < 0.0001. (B) Spleen cells were collected between 9 and 14 dpi, and mitochondrial ROS was assessed in CD11b^+^ cells by measuring MitoSox by flow cytometry after culture with medium or PMA. Data shown are representative of one experiment (n = 4–14 mice per group). Each symbol represents an individual mouse and bars denote group means. Statistical significance was determined by the Mann-Whitney test (^∗^p < 0.05). (C and D) Additional cohorts of uninfected or *C. rodentium*-infected C57BL/6 mice received hemin intraperitoneally (50 μmol kg/body weight per dose) once per day, starting at 3 dpi until 11 dpi and then body weight and survival were monitored. Symbols represent mean ± SEM as a percentage of the initial body weight. Data shown are representative of two independent experiments (n = 4–10 mice per group). (E and F) Additional cohorts of C57BL/6 mice were infected orally with 1 × 10^9^ CFU of the isogenic *C. rodentium fepA* mutant strain or with its control strain DBS120 *C. rodentium* or co-infected with these strains and *P. chabaudi* at day 0. Body weights and survival were monitored. Symbols represent mean ± SEM as a percentage of the initial body weight. Data shown were pooled from two independent experiments (n = 10–33 mice/group).

The central importance of host iron-scavenging pathways in preventing pathogen growth (Skaar, 2010) prompted us to examine whether the cyclical hemolysis caused by *Plasmodium* infection (Easmon, 1884; Stephens, 1901) may be causing systemic pathogen persistence. Systemic inoculation with *C. rodentium* triggers IL-22 secretion that drives the production of hemopexin (HPX), a heme-binding molecule released by the liver that is required for control of systemic *C. rodentium* infection (Sakamoto et al., 2017). We investigated serum HPX levels and found that single infection or co-infection with *P. chabaudi* and/or *C. rodentium* led to robust HPX secretion (Figure S4). We therefore postulated that the high levels of hemolysis caused by *Plasmodium* infection might overwhelm host iron-scavenging pathways. Extensive hemolysis is associated with the accumulation of labile heme in plasma (for review, see Soares and Bozza, 2016), and we found that significantly higher levels of plasma heme were present in co-infected mice than in all other experimental groups (Figure 7A). Elevated heme levels have been associated with many deleterious effects, including impaired phagocytosis, increased ROS production, and cytotoxicity (for review, see Martins and Knapp, 2018). Although phagocytosis was not impaired during co-infection (Figure 6D), we found that, following stimulation with phorbol 12-myristate 13-acetate (PMA), CD11b^+^ splenocytes from co-infected mice produced higher levels of mitochondrial ROS than those from single-infected mice (Figure 7B). Next, to mimic the effects of hemolysis in the absence of malaria, we treated *C. rodentium* single-infected mice with hemin, an iron-containing molecule that is generated from hemoglobin following hemolysis (Schaer et al., 2013). We found that systemic administration of hemin during 3–11 dpi induced weight loss and significant (50%) mortality in *C. rodentium* single-infected mice, albeit not quite as high as the mortality observed in malaria co-infected mice (Figures 7C and 7D).

Gr− enteric pathogens can scavenge environmental iron through siderophores (Khashe and Janda, 1996), small molecules that bind iron-containing molecules and then bind surface receptors on the bacterium to facilitate internalization and iron release (Rutz et al., 1991). *C. rodentium* and pathogenic *E. coli* produce enterobactin, a siderophore, and its receptor FepA to acquire iron and may express functional heme uptake systems (Rutz et al., 1991). To test whether this pathway allows systemic *C. rodentium* to persist during malaria co-infection, we utilized a *C. rodentium* mutant that lacked a key siderophore receptor (Δ*fepA*) as well as the isogenic control *C. rodentium* strain DBS120. Mice co-infected with *P. chabaudi* and *C. rodentium* DBS120 exhibited comparable levels of severe weight loss and mortality to that found with the *C. rodentium* strain used throughout our studies (Figures 7E and 7F). By contrast, mice co-infected with *P. chabaudi* and *C. rodentium* Δ*fepA* had attenuated weight loss and did not exhibit any mortality (Figures 7E and 7F). We found that the *C. rodentium* Δ*fepA* mutant exhibited reduced intestinal colonization compared with its WT DBS120 strain, and this was not affected by co-infection (Figure S5A). Nevertheless, when we examined systemic colonization, we found similar levels of *C. rodentium* in the spleens and livers of DBS120 or Δ*fepA* co-infected mice at 7 dpi (Figure S5B). However, while the DBS120 co-infected cohort showed systemic persistence of *C. rodentium* during the second week of infection, the Δ*fepA* mutant co-infected group completely cleared the systemic bacterial infection (Figure S5B). Taken together, our results show that co-infection led to elevated plasma heme that was associated with increased ROS production from phagocytes and with persistent bacteremia that was dependent on uptake of extracellular iron sources through the FepA receptor. Together, these effects may precipitate severe disease in co-infected animals.

## Discussion

We established a murine model based on co-infection with *P. chabaudi* and *C. rodentium* and found that contemporaneous co-infection produced high levels of morbidity and mortality that were not observed in single-infection cohorts, validating this model for mechanistic interrogation. Co-infection did not alter the course of parasitemia but led to exacerbated bacterial infection in the intestine and prolonged colonization of systemic tissues by *C. rodentium*.

The co-infection model developed here differs significantly from previous models, which have mainly used co-infection with strains of NTS. First, unlike most models of NTS infection, there is no requirement for antibiotic perturbation of the host microbiota, because *C. rodentium* efficiently colonizes the mouse intestine even in the presence of an intact microbiota (for review, see Collins et al., 2014). As intestinal microbiota play a critical role in maintaining local and systemic immune homeostasis (Kabat et al., 2014), antibiotic perturbations may lead to additional immune alterations that affect experimental co-infection with NTS, but that do not reflect the course of clinical disease in humans. Second, many co-infection studies with NTS and malaria bypassed the gut altogether by inoculating NTS systemically, meaning that the potential impact of malaria infection on the local intestinal bacterial infection was largely overlooked (Mooney et al., 2019). However, the natural oral route of *C. rodentium* inoculation allowed us to assess whether concomitant malaria infection had an impact on intestinal infection. Indeed, we found that malaria co-infection led to increased bacterial colonization of the intestinal epithelium and to increased barrier permeability. The observation of increased gut permeability is consistent with previous reports in mouse models of *Plasmodium* infection (Chau et al., 2013; Potts et al., 2016) and has also been reported in humans with malaria (Wilairatana et al., 1997). This could explain why normally self-limiting local infections with extracellular intestinal pathogens exhibit an increased preponderance to translocate and colonize systemic tissues during malaria infection. A third key difference is that NTS rapidly invades across the mouse gut, causing an acute and lethal systemic infection (de Jong et al., 2012; Mooney et al., 2014) and making it very difficult to assess the influence of co-infection on mortality. By contrast, *C. rodentium* elicits only mild disease and no mortality (Mundy et al., 2005), allowing changes in disease severity and mortality to be tracked during co-infection. Finally, the *C. rodentium* co-infection model provides a tractable system to investigate how malaria predisposes to exacerbated mortality during co-infection with Gr− extracellular bacterial pathogens, which have distinct mechanisms of virulence to NTS (Collins et al., 2014).

Nevertheless, studies using co-infection of malaria and NTS have highlighted several potential mechanisms through which malaria infection may impair control of an intracellular Gr− bacterial pathogen. These studies have focused on impaired function of phagocytes, particularly macrophages and neutrophils. The hemolysis and anemia that are triggered by malaria infection lead to the release of heme that is taken up by phagocytes and detoxified through the actions of the enzyme HO-1, which they upregulate during the infection (Cunnington et al., 2012). However, increased HO-1 expression has also been associated with impaired granulocyte mobilization from the bone marrow and with reduced oxidative burst capacity in neutrophils and macrophages (Cunnington et al., 2011; Mitterstiller et al., 2016). In addition, malaria infection also leads to increased production of the anti-inflammatory cytokine IL-10, resulting in reduced activation of mononuclear phagocytes (Lokken et al., 2014, 2018). Together, these mechanisms blunt phagocytic killing pathways, leading to increased survival of intracellular NTS. As an extracellular pathogen, *C. rodentium* does not possess specialized virulence mechanisms that permit intracellular survival in phagocytic compartments, although efficient phagocyte responses are required for bacterial clearance (Collins et al., 2014). However, when we evaluated phagocyte responses in *P. chabaudi* and *C. rodentium* co-infected mice, we did not find any impairment in mobilization or accumulation of monocyte and granulocyte populations. Moreover, we also observed that phagocytosis and killing of *C. rodentium* were not impaired in co-infected mice, pointing toward an alternative mechanism being responsible for the failure to clear systemic bacteria.

We next hypothesized that hyperactivation of pro-inflammatory pathways was driving mortality in co-infected mice. This possibility was suggested by a previous study that showed that *P. chabaudi*-infected mice were hypersusceptible to LPS-triggered septic shock (Ataide et al., 2014; Franklin et al., 2009). When *P. chabaudi*-infected mice were challenged systemically with LPS, there was rapid onset of lethal endotoxic shock that was dependent on inflammasome-mediated IL-1β secretion (Ataide et al., 2014; Franklin et al., 2009; Ataide et al., 2014). However, we found that almost all pro-inflammatory cytokines, including TNF and IL-6, were significantly reduced in co-infected mice relative to the levels found in *P. chabaudi* single-infected mice. Although there was a small increase in circulating IL-1β in co-infected mice, these levels remained very low (<50 pg/mL), especially compared with those reported in *P. chabaudi*-infected mice challenged with systemic LPS (>4,000 pg/mL; Ataide et al., 2014). In addition, we also found that *Asc*^*−/−*^ mice, *caspase-1*^*−/−*^ mice, and *Nlrp3*^*−/−*^ mice exhibited comparable mortality to WT controls following co-infection with *P. chabaudi* and *C. rodentium*, as did mice lacking the LPS sensors *Tlr4* or *caspase-11*. Taken together, our results indicate that the mortality induced following co-infection with *P. chabaudi* and *C. rodentium* was not due to hyperactivation of innate immune pathways or LPS-triggered endotoxic shock.

Nutritional immunity—the regulation of essential nutrient and co-factor availability—is gaining increasing re-appreciation as a crucial protective mechanism against pathogenic infection (Sakamoto et al., 2017). Iron is essential for all living organisms, and several pathways operate to restrict iron availability during pathogenic infection (Cassat and Skaar, 2013). The situation becomes even more complex in the context of malaria infection, where severe anemia poses a major problem, but the relationship between iron availability and malaria infection remains incompletely understood (for review, see Spottiswoode et al., 2014). Indeed, somewhat counterintuitively, iron deficiency appears to protect from severe malaria, and whether iron supplementation is beneficial or detrimental in malaria-exposed populations has been hotly debated (Gwamaka et al., 2012; Jonker et al., 2012; Muriuki et al., 2019; Murray et al., 1978; Sazawal et al., 2006).

During infection, the iron regulatory hormone hepcidin is upregulated, which decreases serum iron levels by reducing iron absorption in the gut and promoting iron accumulation in macrophages, with the latter effect being proposed to increase susceptibility to NTS (Cunnington et al., 2011; van Santen et al., 2013). Similarly, co-infection of mice with *Plasmodium yoelii* and *Salmonella* Typhimurium led to increased ferritin expression in macrophages, suggesting that increased iron availability promotes intracellular pathogen growth (Lokken et al., 2014, 2018). Thus, together with the effects of HO-1 noted above, it is clear that iron availability and metabolism strongly affect susceptibility to co-infections with NTS. However, recent work has also highlighted the importance of restricting iron availability during systemic infection with extracellular bacterial pathogens, such as *C. rodentium*. Repeated intravenous inoculation with *C. rodentium* induced an early IL-22 response that triggered secretion of the host heme-scavenging molecule HPX by the liver (Sakamoto et al., 2017). Although WT mice were highly resistant to systemic *C. rodentium* infection, mice lacking the IL-22-HPX axis succumbed to infection (Sakamoto et al., 2017). However, we found that either single *P. chabaudi* infection or co-infection with *C. rodentium* induced robust HPX secretion, indicating that this protective axis was intact.

We therefore posited that the high levels of hemolysis triggered during *P. chabaudi* infection may overwhelm host iron-scavenging pathways. Hemolysis releases hemoglobin into plasma, which in turn releases labile heme, a highly reactive molecule that can interact with a range of host molecules and cells to cause toxic and immunomodulatory effects (Martins and Knapp, 2018). We found that plasma heme was selectively elevated in co-infected mice, even though parasitemia levels, RBC counts and hemoglobin levels were comparable with those observed in single *P. chabaudi*-infected mice. This suggests that concurrent bacteremia alters some of the host responses that are normally triggered to scavenge and catabolize labile heme during malaria-induced hemolysis, and further studies are required to identify the molecular mechanisms responsible. Indeed, our current knowledge of how labile heme is scavenged and/or signals during infection-associated episodes of extensive hemolysis remains incomplete (Martins and Knapp, 2018; Soares and Bozza, 2016). We also mimicked extensive hemolysis by administering hemin, a heme-containing molecule generated upon hemolysis, to mice infected with *C. rodentium* and found that this resulted in significant levels of mortality. There are several ways in which excessive heme may contribute to mortality in co-infected mice. Heme has been reported to activate innate immune receptors, including TLR4 (Belcher et al., 2014; Figueiredo et al., 2007) and the NLRP3 inflammasome (Dutra et al., 2014; Li et al., 2014). However, our results showing that co-infected mice do not exhibit cytokine storm and that neither TLR4 nor NLRP3 is required for lethality suggest that this is not the key mechanism. Alternatively, elevated heme levels have been implicated in hemolysis-associated immunosuppression and in altered macrophage and neutrophil functions, including phagocytosis, cytotoxicity, and increased ROS production (Martins and Knapp, 2018). As noted above, during malaria and NTS co-infection, heme induction of the cytoprotective HO-1 response was associated with impaired granulocyte killing and enhanced NTS growth (Cunnington et al., 2011). Similarly, it was reported that increased heme levels exacerbated mortality in a model of *E. coli*-induced acute sepsis and that this was due to heme-mediated inhibition of phagocytosis (Martins et al., 2016). However, as described above, we found no defect in either accumulation or phagocytosis by monocytic and granulocytic CD11b^+^ cells in the co-infected mice, suggesting that additional effects of heme may be driving the mortality phenotype. It is therefore pertinent that we observed increased potential for mitochondrial ROS production from CD11b^+^ cells from co-infected mice, suggesting that some altered phagocyte responses could contribute, as ROS generation has been reported to play a key role in heme-driven cytotoxicity (Dixon and Stockwell, 2014; Fortes et al., 2012). Furthermore, elevated plasma heme played a critical role in the pathogenesis of acute polymicrobial sepsis in mice, where heme synergized with other cytotoxic molecules to trigger cell death in a ROS-dependent manner (Larsen et al., 2010). Heme has also been reported to induce the formation of neutrophil extracellular traps (NETs) through a ROS-dependent mechanism (Chen et al., 2014). NETs are complexes of chromatin and neutrophil granule contents that act to trap and kill bacterial pathogens, but they can also mediate tissue pathology (Chen et al., 2014). However, whether ROS-driven cytotoxicity or NETs contribute to pathogenesis and mortality during malaria/bacterial co-infections remains to be established.

Elevated heme levels could also provide a potential source of iron to facilitate bacterial persistence. We utilized an isogenic mutant strain of *C. rodentium* lacking FepA, a ferric enterobactin transport ATP-binding protein that mediates uptake of bacterial siderophores complexed with iron-containing molecules (Rutz et al., 1991). We demonstrated that co-infection with *P. chabaudi* and *C. rodentium* Δ*fepA* did not induce any mortality. One partial caveat was that the *C. rodentium* Δ*fepA* mutant exhibited reduced intestinal colonization relative to the WT *C. rodentium*. This defect could not be overcome by increasing the initial infection dose (data not shown) and was not completely unexpected, because *C. rodentium* must compete with endogenous microbiota for environmental nutrients, including iron. Nevertheless, we found that the *C. rodentium* Δ*fepA* mutant was able to translocate and reach systemic tissues in comparable levels to the WT *C. rodentium* during the first week of co-infection. Crucially however, the inability to efficiently acquire iron meant that the *C. rodentium* Δ*fepA* mutant was unable to persist systemically and cause mortality. Although this is the simplest explanation for our observations, it is possible that iron acquisition also affects expression of *C. rodentium* systemic virulence factors and that this may also have contributed to the reduced pathology in mice co-infected with the *C. rodentium* Δ*fepA* mutant. It is also worth noting that although mechanisms of iron acquisition are likely to differ between extracellular pathogens such as *C rodentium* and *E. coli* and intracellular pathogens such as NTS, the fepA gene is also present in many *Salmonella* strains (Tan et al., 2019), and the potential contribution of siderophore-mediated iron acquisition during NTS/malaria co-infection remains to be ascertained.

Overall, we postulate that co-infection with *C. rodentium* and *P. chabaudi* triggers sustained elevation of plasma heme that fuels a vicious cycle of bacteremia and organ damage that eventually proves fatal. Malaria infection leads to numerous consequences that enhance susceptibility to concomitant bacterial infections, several of which involve host iron metabolic pathways. A better understanding of the regulation of host iron metabolism during malaria infection is not only important for devising improved treatment strategies for severe anemia but also for improved management of bacterial co-infections. Such strategies may have relevance beyond malaria, for example, a recent study of a large cohort of sepsis patients reported that high serum iron levels were associated with an increased risk of mortality, with the vast majority of these infections attributable to extracellular bacterial pathogens (Lan et al., 2018).

## STAR★Methods

### Key Resources Table

**Table:** 

REAGENT or RESOURCE	SOURCE	IDENTIFIER
**Antibodies**		

anti-CD11b-APC	eBioscience (ThermoFisher)	Clone M1/70 Cat# 17-0112-82; RRID: AB_469343
anti-CD45-APC-eFluor780	eBioscience (ThermoFisher)	Clone 104 Cat# 470454-82; RRID: AB_1272175
anti-CD11b-PE	eBioscience (ThermoFisher)	Clone M1/70 Cat# 12-0112-82: RRID: AB_2734869
anti-CD11b-PE-Cy7	eBioscience (ThermoFisher)	Clone M1/70 Cat# 25-0112-82; RRID: AB_469588
anti-Ly6C-eFluor450	eBioscience (ThermoFisher)	Clone HK1.4 Cat# 48-5932-82; RRID: AB_10805519
anti-Ly6G-PE	eBioscience (ThermoFisher)	Clone 1A8 Cat# 12-9668-82; RRID: AB_2572720
rabbit anti-mouse hemopexin	Abcam	Cat# ab90947; RRID: AB_2049748
goat anti-rabbit IgG antibody	Abcam	Cat# ab205718; RRID: AB_2819160

**Bacterial and Virus Strains**		

Citrobacter rodentium	Gad Frankel, Imperial College, London, UK	ICC169
GFP-Citrobacter rodentium	Gad Frankel, Imperial College, London, UK	N/A
*Citrobacter rodentium* Δ*fepA mutant*	Dr. Gabriel Núñez, University of Michigan Medical School, USA.	N/A
*C. rodentium* DBS120	Dr. Gabriel Núñez, University of Michigan Medical School, USA.	N/A

**Chemicals, Peptides, and Recombinant Proteins**		

Hemin	Sigma-Aldrich	Cat# 51280
Naladixic Acid	Sigma-Aldrich	Cat# N4382
Paraformaldehyde	Sigma-Aldrich	Cat# 33220
Sybr Green	Invitrogen	Cat# S-7567
Giemsa Stain	Sigma-Aldrich	Cat# GS500

**Critical Commercial Assays**		

Cytometric Bead Array Mouse Th1/Th2/Th17	BD bioscience	Cat# 560485
Mouse IL-1b ELISA	R&D Systems	Cat# DY401E
Live/Dead Fixable cell stain	ThermoFisher	Cat# L34957
ALT kinectic Kit	Bioclin	Cat# K049-1
QuantiChrom Heme Assay kit	BioAssay Systems	Cat# DIHM-250
MitoSOX Red mitochondrial superoxide	Invitrogen	Cat# M36008

**Experimental Models: Organisms/Strains**		

Mouse: C57BL/6J	The Jackson Laboratory	JAX 000664
Mouse: TLR4−/−	The Jackson Laboratory	JAX 029015
Mouse: NLRP3−/−	The Jackson Laboratory	JAX 021302
Mouse: AIM2−/−	The Jackson Laboratory	JAX 013144
Mouse: Caspase1−/−	The Jackson Laboratory	JAX 032662
Mouse: Caspase 11−/−	Dr. Vishva Dixit from Genentech (San Francisco, CA)	N/A
Mouse: ASC−/−	Dr. Vishva Dixit from Genentech (San Francisco, CA)	N/A
*Plasmodium chabaudi: P. chabaudi chabaudi AS* strain	Dr. D’Imperio-Lima, Universidade de São Paulo; Institute Pasteur	N/A

**Software and Algorithms**		

BD FACSuite software	BD Bioscience	https://www.bdbiosciences.com/en-us
FCAP Array software	BD Bioscience	https://www.bdbiosciences.com/en-us
FlowJo V10.0.2	Tree Star	https://www.flowjo.com/
GraphPad PrismV8.0	GraphPad-Software	https://www.graphpad.com/
Adobe Illustrator	Adobe	https://www.adobe.com/

### Resource Availability

#### Lead Contact

Further information and requests for resources and reagents should be directed to and will be fulfilled by the Lead Contact, Lis Ribeiro do Valle Antonelli (lis.antonelli@fiocruz.br) and Kevin Maloy (kevin.Maloy@glasgow.ac.uk).

#### Materials Availability

This study did not generate new unique reagents.

#### Data and Code Availability

The published article includes all datasets generated or analyzed during this study. This study did not generate any unique codes.

### Experimental Model and Subject Details

#### Mice and treatments

C57BL/6J, mice were purchased from Jackson Laboratories. The fully backcrossed knockout mice, ASC^**−/−**^, NLRP3−/− and Caspase 11−/− mice were provided by Dr. Vishva Dixit from Genentech (San Francisco, CA). AIM2^−/−^ mice were originally provided by Katherine A. Fitzgerald (Worcester, MA), Caspase-1 knockout mice used in this work was provided by Dr. Devi Kanneganti Thirumala from Jude Children’s Research Hospital (Menphis, TN), and TLR4−/− mice were obtained from Dr. Shizuo Akira from Osaka University (Osaka, Japan). Mice were bred in isolated conditions receiving sterile water and food at animal facilities from Oswaldo Cruz Foundation or University of Oxford. We used sex-matched mice between 8–12 weeks of age. Procedures were conducted in accredited animal facilities under the project licenses PPL30/3423 and LW36/14 authorized respectively by the UK Home Office Animal Procedures Committee and the Council of Animal Experimentation of Oswaldo Cruz Foundation. Hemin (Sigma) was administered by i.p. injection (50 μmol/kg body weight/dose) once per day, starting at 3 until 11 dpi.

#### Bacteria

A single *C. rodentium* colony was transferred to nalidixic acid-supplemented LB broth and grown overnight to saturation. The next day, the culture was diluted to an optical density of 0.1 and grown to log phase before harvest by centrifugation and resuspension in phosphate-buffered saline (PBS). Mice were gavaged with 200 μL of PBS containing ∼10^9^ *C. rodentium* (ICC169). Mice were then weighed every day and culled if weight loss exceeded 20% of starting weight. To measure the *C. rodentium* load, tissues were weighed and then homogenized in 600 μL of PBS. Serial dilutions of tissue lysates were plated on nalidixic acid (Sigma; final concentration 50 μg/ml) agar plates and then incubated at 37°C overnight before counting colonies. The number of colonies were normalized to the weight of the tissue (CFU/g). For *in vitro* experiments we used GFP-*C. rodentium* (nalidixic acid and chloramphenicol resistant). *C. rodentium* Δ*fepA* mutant was generated using the λ-Red recombinase system previously described by (Datsenko and Wanner, 2000). Briefly, a PCR product containing the kanamycin resistance cassette flanked by homologous extensions to regions adjacent to the target gene (ID: CBG87369) was transformed into *C. rodentium* DBS100 containing the vector pKD46, a temperature sensitive plasmid expressing the phage λ-Red recombinase. Mutants were isolated on LB plates as kanamycin-resistant colonies, and the pKD46 plasmid was then removed by growing the isolated mutants at 42°C. Kanamycin resistant (50 μg/ml) strains *C. rodentium* Δ*fepA* and its isogenic wild-type *C. rodentium* DBS120 (pCRP1:Tn5) were cultured and inoculated as previously described for *C. rodentium*.

#### Parasite infection

C57BL/6 RBC infected with *Plasmodium chabaudi chabaudi AS* strain were stored in liquid nitrogen and thawed and passed into WT mice once a week. Mice were injected i.p. with 10^6^ infected RBC and parasitaemia followed every day using Giemsa-stained (Sigma) thin blood smears and SYBR Green I (Invitrogen) stained as previously described (Izumiyama et al., 2009). The RBC numbers and HGB levels were determined in heparinized blood (Scil Vet ABC hematology analyzer). Serum ALT activity was performed using a kinetic test (Bioclin).

### Method Details

#### Assessment of intestinal inflammation

Mice were euthanized at the indicated time points during infection whereupon tissue sections were cut and fixed in buffered 10% formalin. Sections were cut and stained with hematoxylin and eosin (H&E). Analysis was performed as described (Song-Zhao et al., 2014). Briefly, inflammation was graded semiquantitatively on a scale from 0-3, for five criteria; (a) epithelial hyperplasia and goblet cell depletion, (b) lamina propria leukocyte infiltration, (c) submucosa edema and infiltration, (d) area of tissue affected, and (e) markers of severe inflammation, including crypt abscesses and ulceration. Scores for individual criteria were totaled for an overall inflammation score between 0 and 15.

#### Cytokine measurements

IL-1b were assessed in serum using commercially available ELISA Duoset (R&D Systems). TNF, IL-6, IFN-γ, IL-17, IL-10 levels were assessed in the serum and organ explant culture using the Cytometric Bead Array Mouse Th1/Th2/Th17 according to the manufacturer’s instructions (BD bioscience). Samples were acquired using BD FACSVerse and BD FACSuite software, analyzed by FCAP Array software. To perform organ explant culture, fragments of colon and cecum were weighed, washed in antibiotic supplemented PBS and then incubated 37°C overnight in complete RPMI (GIBCO). Supernatants were harvested for cytokine measurement.

#### C. rodentium phagocytosis and killing assays

We assessed *ex vivo* phagocytosis and killing of GFP*-C. rodentium* in a gentamicin protection assay and quantified by flow cytometry staining with CD11b-APC (eBioscience). Spleens were collected from uninfected and infected mice and RBC lysis were performed. After washing, cells were plated at 4x10^5^ cells/well in RPMI without antibiotics. GFP-*C. rodentium* were added to culture at a multiplicity of infection (MOI) of 10 bacteria:1 splenocyte and incubated at 37°C and 5% CO2. As negative control, uninfected cells were incubated in parallel for each animal. Plates were incubated for 1 hour before addition of gentamicin (100ug/ml) to kill extracellular bacteria. Phagocytosis was determined after 60min and alternatively, to assess bacterial killing, cells were washed twice with warm medium, then reincubated for 3h in medium containing 10 μg/ml gentamicin to prevent extracellular growth of GFP*-C. rodentium.*

#### Heme and ROS measurement

Plasma samples were used to perform a Quantichrom Heme assay (BioAssay Systems) according to the manufacturer’s instructions. Mitochondrial ROS was assessed by MitoSox red mitochondrial superoxide indicator (Invitrogen) following the manufacturer’s instructions. Briefly, splenocytes were washed with HBSS and incubated during 10min at 37°C with 5 μM MitoSox in medium alone or with PMA (10ng/ml). Cells were washed with warm PBS and incubated with monoclonal antibody anti-CD11b-PE (eBioscience). Acquisitions were performed using a FACS LSR Fortessa flow cytometer (Becton Dickinson) and data were analyzed using FlowJo V10.0.2 (Tree Star).

#### Imunophenotyping

Splenocytes were obtained from uninfected and infected animals and after RBC lysis, cells were washed in PBS, incubated with Live/Dead Fixable cell stain (ThermoFisher) for cell death exclusion, and then, with the following monoclonal antibodies: anti-CD45-APC-Cy7, anti-CD11-b-PE-Cy7, anti-Ly6C-eFluor450 and anti-Ly6G-PE (eBioscience). Samples were washed and fixed with paraformaldehyde 4% (Sigma-Aldrich) and data were acquired on a FACS LSR Fortessa flow cytometer (Becton Dickinson). Data were analyzed with FlowJo V10.0.2 (Tree Star). A forward scatter area (FSC-A) versus forward scatter height (FSC-H) gate was used to remove doublets. Viable leukocytes were selected using a Live/Dead versus CD45 gate. Expression of Ly6C and Ly6G was used to define neutrophils (Ly6C^int^Ly6G^high^) and monocytes (Ly6C^high^Ly6G^int^) within CD11b^+^ cells. GraphPad PrismV8.0 (GraphPad-Software) was used for graphic representation.

#### Clinical scale used to determine disease severity in mice

**(**1) no signs, (2) ruffled fur and/or abnormal posture and/or minor weight loss (< 15%), **(**3) lethargy and/or moderate weight loss (≥20%), **(**4) reduced response to stimulation and/or ataxia and/or respiratory distress/hyperventilation, (5) prostration and/or paralysis and/or convulsions and/or severe weight loss (> 25%), (6) Death. The humane endpoint was defined as mice reached stage 3 or stage 4. In these stages, mice were called moribund and were euthanized.

#### Imunoblotting

Diluted plasma samples were boiled in Laemmli sample buffer and equivalent amounts of 0.5 μL of plasma were separated by SDS-PAGE and transferred onto membranes by electro-blotting. The blot was immunoblotted with rabbit anti-mouse hemopexin (Abcam). Protein bands were revealed with goat anti-rabbit IgG antibody (Abcam) and enhanced chemiluminescent substrate (Thermo Scientific).

### Quantification and Statistical Analysis

For weight curves p values were determined by two-way ANOVA and for survival graphs were used Mantel-Cox. For other experiments, p values were determined by nonparametric Mann–Whitney test, or by Wilcoxon paired test. Differences were considered statistically significant when p < 0.05 (^∗^p < 0.05; ^∗∗^p < 0.01; ^∗∗∗^p < 0.001, ^∗∗∗∗^p < 0.0001). Statistics were calculated using GraphPad Prism 8 software. The statistical tests applied as well as the number of experimental repeats are specified in the figure legends.

## Supplemental Information

Document S1. Figures S1–S5

Document S2. Article plus Supplemental Information
