# Supplementary material for: Disrupted Iron Metabolism and Mortality during Co-infection with Malaria and an Intestinal Gram-Negative Extracellular Pathogen
Source: Cell Rep. 2021 Jan 12;34(2):108613. doi: 10.1016/j.celrep.2020.108613 (PMC8655499; doi:10.1016/j.celrep.2020.108613)
Supplement: Document S1. Figures S1–S5 [file mmc1.pdf]

Cell Reports, Volume *34*

Supplemental Information

Disrupted Iron Metabolism and Mortality during Co-infection with Malaria and an Intestinal Gram-Negative Extracellular Pathogen

Luara Isabela dos Santos, Thais Abdala Torres, Suelen Queiroz Diniz, Ricardo Gonçalves, Gustavo Caballero-Flores, Gabriel Núñez, Ricardo Tostes Gazzinelli, Kevin Joseph Maloy, and Lis Ribeiro do V. Antonelli

SUPPLEMENTAL

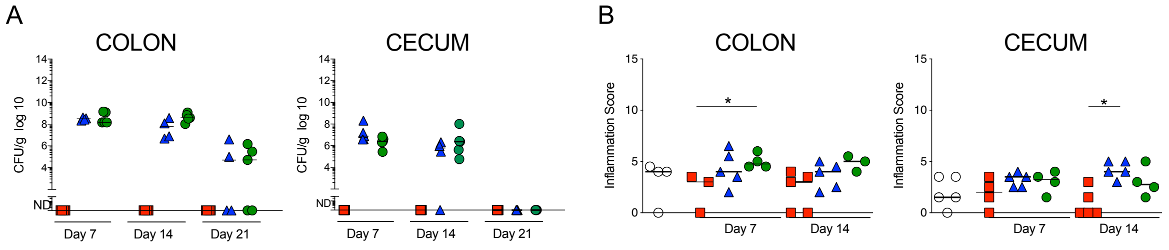

Figure S1. Clearance of *C. rodentium* in surviving co-infected mice, Related to Figure 3. C57BL/6 mice were infected intraperitoneally with 1x10^{6} *P. chabaudi*-RBC, or orally with 1x10^{9} *C. rodentium,* or co-infected with both *C. rodentium and P. chabaudi* at day 0. At the indicated time points, colon and cecum samples were harvested for further analyses. (A) *C. rodentium* colonization was determined by plating homogenized tissue samples on agar containing naladixic acid. Each symbol represents an individual mouse and bars denote group means. Data shown are representative of three independent experiments (n=4-5 mice per group for each time point). (B) Tissue samples were fixed, then stained with hematoxylin/eosin and inflammation scores were evaluated. Each symbol represents an individual mouse and bars denote group means. Results are representative of two independent experiments (n=4-5 mice per group for each time point). Statistical significance was determined by Mann-Whitney test *p<0.05.

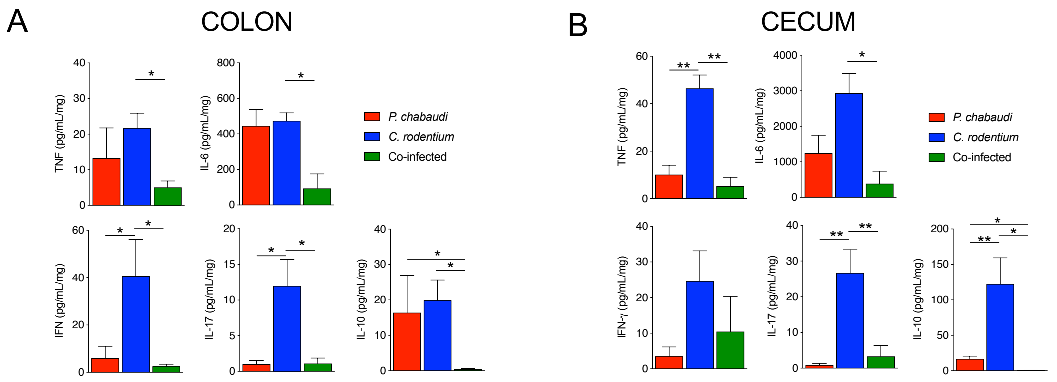

Figure S2. Co-infection decreases intestinal cytokine responses compared to single infection with *C. rodentium*, Related to Figure 4. C57BL/6 mice were infected intraperitoneally with $1x10^6$ *P. chabaudi*-RBC, or orally with $1x10^9$ *C. rodentium,* or co-infected with both *C. rodentium and P. chabaudi* at day 0. (A) Colon and (B) cecum tissue explants were harvested between 9 and 14 dpi and cultured overnight. Supernatants were collected and cytokine levels were and cytokine levels assayed using cytometric bead assay. Bars represent group means ± SEM. Data shown are representative of two independent experiments (n=4-5 mice per group). Statistical significance was determined by Mann-Whitney test, *p<0.05; **p<0.01.

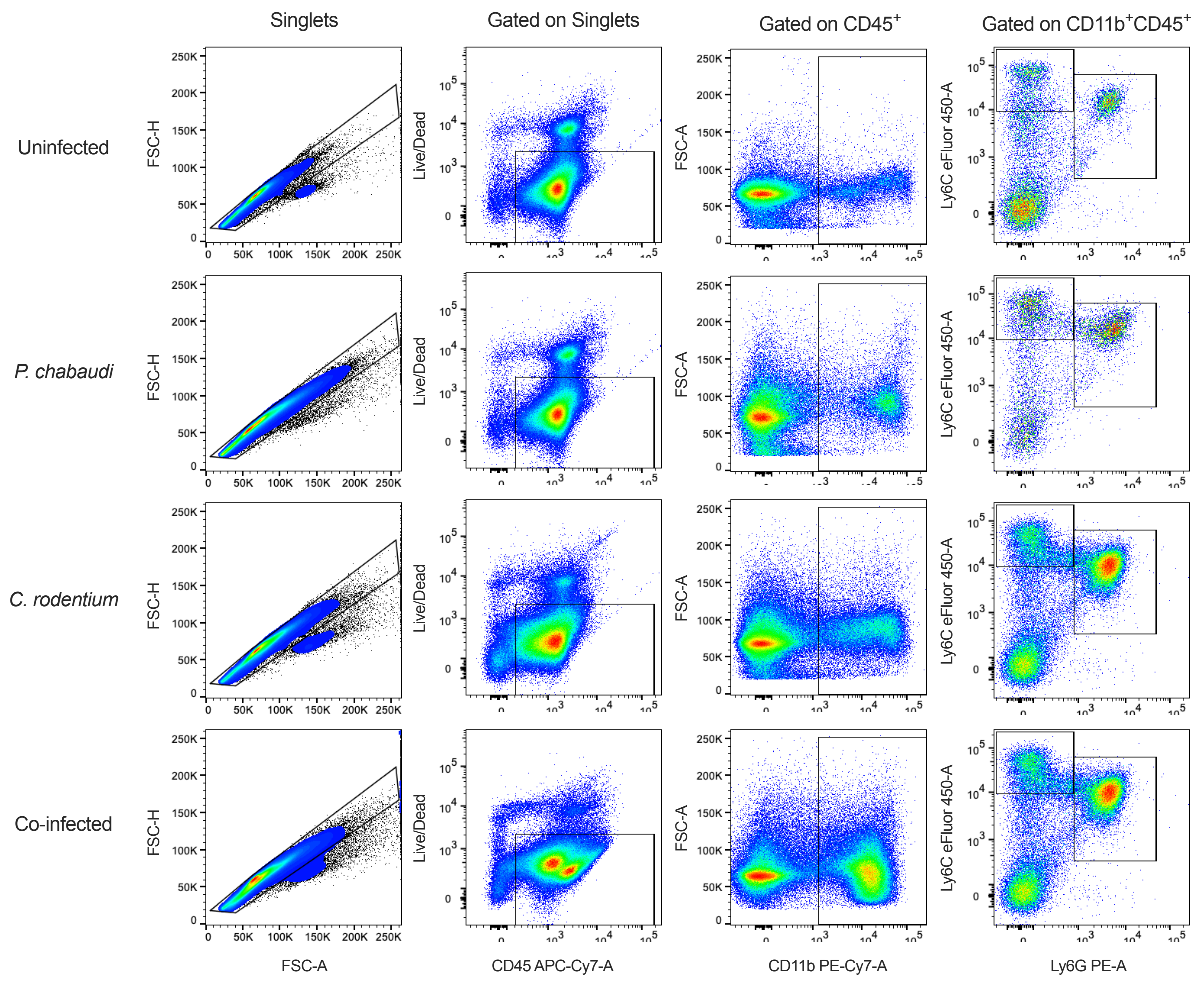

Figure S3. Myeloid cell immunophenotyping strategy, Related to Figure 6. Spleen cell suspensions were analysed using flow cytometry**.** Singlet cells were gated using forward scatter area (FSC-A) versus forward scatter height (FSC-H). Viable luekocytes cells were gated using a Live/Dead stain in combination with anti-CD45, then myeloid cells identified using anti-CD11b antibodies. Expression of Ly6C and Ly6G on $CD45^{+}C11b^{+}$ cells was used to define neutrophils ($Ly6C^{int}Ly6G^{high}$) and monocytes ($Ly6C^{high}Ly6G^{int}$).

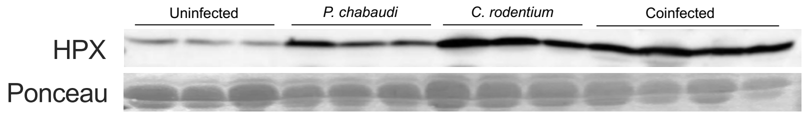

Figure S4. Co-infection leads to comparable induction of hemopexin (HPX) as single infection with either *P. chabaudi* or *C. rodentium,* Related to Figure 7. C57BL/6 mice were infected intraperitoneally with 1x10^6 *P. chabaudi*-RBC, or orally with 1x10^9 *C. rodentium,* or co-infected with both *C. rodentium and P. chabaudi* at day 0. Plasma samples from C57Bl/6 mice (n = 3 to 4 per group) were collected between 9 and 14 dpi and immunoblotted with antibodies against HPX. Staining of gels with Ponceau S as a loading control is also shown.

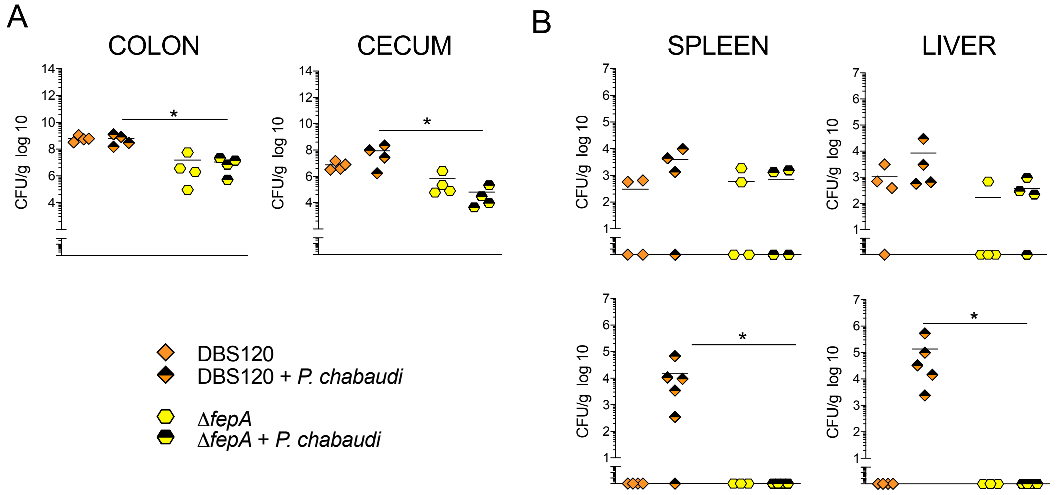

Figure S5. An isogenic *C. rodentium* Δ*fepA* strain shows reduced intestinal colonization but comparable systemic translocation during co-infection with *P. chabaudi*, Related to Figure 7. Cohorts of C57BL/6 mice were infected orally with 1x10^9 CFU of the isogenic *C. rodentium fepA* mutant strain or with its control strain DBS120 *C. rodentium,* or co-infected with these strains and *P. chabaudi* at day 0. (A) Colon and cecum were harvested at 7 dpi to measure intestinal colonization. (B) spleen and liver were harvested at day 7 (top panels) and between 9 and 14 (bottom panels) dpi to quantify systemic bacterial loads. Bacteria colonization was determined by plating homogenized tissues in agar containing kanamycin. Each symbol represents an individual mouse and bars denote group means. Results are representative of two independent experiments (n=4-6 mice per group for each time point). Statistical significance was determined by Mann-Whitney test, *p<0.05.
